# Influence of Hf Doping on the Oxygen Behaviors on ZrCo(110) Surface Using First-Principles Calculation

**DOI:** 10.3390/ma17102424

**Published:** 2024-05-17

**Authors:** Ruijun Qian, Habibullah, Meitong Ye, Wanglai Cen, Chaoling Wu

**Affiliations:** 1 College of Materials Science and Engineering, Sichuan University, Chengdu 610064, China; qrj1430757297@163.com (R.Q.); 18328300781@163.com (M.Y.); 2 Institute of New-Energy and Low-Carbon Technology, Sichuan University, Chengdu 610064, China; habibullah@stu.scu.edu.cn (H.); cenwanglai@scu.edu.cn (W.C.)

**Keywords:** ZrCo, Hf doping, oxygen poisoning, first principles, diffusion

## Abstract

ZrCo alloy is easily poisoned by impurity gases such as O_2_, CO, and CO_2_, resulting in a deterioration in hydrogen storage performance. In this study, we conducted a comprehensive investigation into the adsorption and dissociation characteristics of oxygen on the ZrCo(110) surface using first-principles calculations. Previous studies indicated that the anti-disproportionation properties of ZrCo alloy can be significantly improved by Hf substitution, but the effect of Hf doping on the anti-poisoning properties has not been reported. We also examined the effect of Hf doping on the adsorption, dissociation, and diffusion characteristics of oxygen. It is found that on the ZrCo(110) surface, O_2_ molecules are easily dissociated and then stably adsorbed at the hollow site. Oxygen atoms will fill the surface preferentially and then diffuse inward. The doping of Hf has an insignificant impact on the adsorption or dissociation behavior of oxygen in comparison to the pure ZrCo surface. However, a notable observation is that the doping of Hf resulted in a reduction in the diffusion barrier for oxygen from the surface to the subsurface by 0.61 eV. Consequently, our study suggests that doping Hf is not an advisable strategy for improving the ZrCo(110) surface’s resistance to O_2_ poisoning because of improved oxygen permeability.

## 1. Introduction

In recent years, nuclear fusion energy has gained prominent attention as an ideal future clean energy source because of its advantages of high energy density, abundant fuel resources, and minimal environmental impact [1]. Safe and efficient transportation and storage of tritium is particularly important to ensure the smooth progress of thermonuclear fusion reactions [2]. As the isotope of hydrogen, tritium has similar chemical properties to hydrogen. Therefore, hydrogen is used to simulate tritium in many tritium storage experiments for safety and cost considerations. The capacity of uranium (U) to absorb tritium at room temperature with a low equilibrium pressure for hydrogen absorption and desorption at moderate temperatures positions it as a good candidate for hydrogen and its isotope storage [3]. However, the scarcity, high price, pulverization, radioactivity, and spontaneous combustion of uranium limit its further application. ZrCo alloy has the advantages of low equilibrium hydrogen pressure, high hydrogen absorption capacity, zero radioactivity, and low spontaneous combustion [4,5,6]. Therefore, it is considered one of the most likely alternatives to replace uranium as a new hydrogen (tritium) storage material in the International Thermonuclear Experimental Reactor (ITER) project [7,8].

Although the ZrCo alloy shows good hydrogen storage performance, it is prone to disproportionation and gaseous impurity poisoning. The hydrogen-induced disproportionation during the absorption and desorption cycles leads to a serious decline in the storage capacity of ZrCo alloy [9,10]. The mechanisms of the disproportionation of ZrCo hydride [11,12] and the doping effect on the disproportionation resistance of the ZrCo alloy [13,14,15] have been studied systematically in recent years.

Previous studies have shown that ZrCo alloy is easily poisoned by impurities such as CO, CO_2_, N_2_, and O_2_, resulting in a decline in hydrogen absorption rate and hydrogen absorption capacity [4,16,17]. The decline may be attributed to the formation of carbides, nitrides, and oxides on the alloy surface, which hinders the adsorption and dissociation of hydrogen. For example, Penzhorn et al. [4] found that ZrCo powders were completely oxidized to ZrCoO_3_ at 630 °C in an O_2_ atmosphere, completely losing their hydrogen storage capacity. From surface analytical investigations of ZrCo after exposure to CO, Glasbrenner et al. [16] found that the reaction mainly occurred between CO and the Co component, decomposing CO to graphite and CO_2_, with the formation of carbides. Few studies have been carried out to improve the anti-poisoning properties of ZrCo alloys [18], but many studies have shown that the anti-poisoning properties of Zr-based alloys can be improved by surface modification [19] and doping [17,20,21]. Zhang et al. [19] deposited Pd-Ag coating on different Zr-based Lave phase alloys by the electroless plating technique, which significantly elevated the poisoning resistance of alloys to 1 vol.% air gaseous impurities at different temperatures. Yang et al. [21] investigated the effects of doping Hf, Pd, and Ti on the anti-poisoning properties of the ZrV_2_ alloy. The results showed that the doping of Pd and Hf resulted in a good anti-poisoning effect for the alloys.

Over the past decade, numerous theoretical and computational studies have been carried out to solve the disproportionation and poisoning issues of ZrCo. However, relatively limited research has been carried out on the microscopic poisoning mechanisms by impurity gases, including O_2_ poisoning behaviors on the surface of the alloys, and the anti-poisoning mechanisms remain inadequately elucidated.

Existing studies have shown that Hf doping can improve the anti-poisoning performance of ZrV_2_ [21], but whether Hf doping is beneficial for the anti-poisoning performance of ZrCo is still unclear. Therefore, in this study, we thoroughly investigated the adsorption, dissociation, and diffusion of oxygen on the ZrCo(110) surface using DFT calculations. We directly analyzed the effect of Hf doping on the adsorption, dissociation, and diffusion characteristics of oxygen, with the aim of finding out whether the doping of Hf is conducive to resistance to O_2_ poisoning. The interactions between the surface atoms are further investigated by the partial density of states, differential charge density, and Bader charge analysis.

## 2. Computational Methods

**Figure 1:**
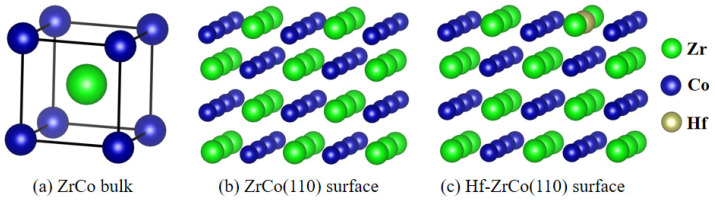
Structures of (**a**) ZrCo bulk, (**b**) ZrCo(110) surface, and (**c**) Hf-ZrCo(110) surface.

DFT calculations were conducted utilizing the Vienna ab initio simulation package (VASP-5.4.4) [22]. Generalized gradient approximation (GGA) of the Perdew–Burke–Ernzerh (PBE) function was employed to model the exchange-correlation interactions, while electronic interactions were described using pseudopotentials with an energy cutoff of 400 eV [23]. It has been established in prior research that the ZrCo(110) surface exhibits the highest stability among ZrCo surfaces [24], hence this surface was selected as the focus of the current study. A 3 × 2 supercell was utilized to simulate the ideal (110) surface, comprising four atomic layers. The introduction of a Hf atom by substituting a Zr atom resulted in the formation of the Hf-doped ZrCo(110) surface, as depicted in Figure 1. The bottom two layers were held fixed during the calculation to simulate the bulk phase, while relaxation of the top two layers simulated the surface. A vacuum space of 15 Å was included to prevent interactions between adjacent slabs. Brillouin-zone integration was performed using Monkhorst–Pack grids of special points [25]. A 4 × 4 × 1 k-point grid was employed for both the pure ZrCo(110) surface and the Hf-doped ZrCo(110) surface. The convergence criteria for the relaxation of each atomic structure included a force of 0.01 eV/Å, while the energy convergence criterion for self-consistent iteration was set to 10^−5^ eV.

The adsorption energy of O_2_/O in all configurations is defined by the following formula [26]:

E_ads_ = E_slab+X_ − E_slab_ − E_X_ (1)

where E_slab+X_ is the total energy of the adsorption system, E_slab_ is the total energy of the pure or doped surface, and E_X_ is the total energy of the isolated O_2_ molecules or O atoms in vacuum.

The Climbing Image Nudge Elastic Band (CI-NEB) method [27] was used to study the minimum energy path (MEP) and the energy barriers of O_2_ molecules dissociation and O atoms diffusion on the ZrCo(110) surface and Hf-doped ZrCo(110) surface. Additionally, further understanding of the behavior of the adsorption systems was obtained by analyzing the partial density of states (PDOS), the differential charge density (DCD), and the Bader charge. These analyses are helpful in understanding the mechanism of surface oxidation.

## 3. Results and Discussion

### 3.1. Structures Relaxation

Chattaraj studied the surface energies of the (100), (110), and (111) ZrCo surfaces and found that the (110) surface is the most stable one [24]. We selected the (110) surface as the focus of our study and optimized the ZrCo bulk, ZrCo(110) surface, and Hf-doped ZrCo(110) surface. The lattice parameter of bulk ZrCo calculated in this study is 3.194 Å, which is in line with the experimental value of 3.196 Å [28]. After structural optimization, there are visible wrinkles on the surfaces due to the variance of the surface atomic coordinate. The wrinkles are described by the following formula [29]:

Δd = (d_rel_ − d_ini_)/d_ini_ × 100% (2)

where d_rel_ is the coordinate or the distance after relaxation and the d_ini_ is the coordinate or the distance before relaxation. The negative values of Δd stand for contraction, with positive values corresponding to expansion.

Table 1 shows the z coordinates of atoms on the ZrCo(110) surface and Hf–ZrCo(110) surface, where first and second represent the first and the second atomic layer, respectively. d_12_(Co-Zr), d_12_(Zr-Co), and d_12_(Hf-Co) refer to the distances between surface Co and subsurface Zr, surface Zr and subsurface Co, and surface Hf and subsurface Co, respectively.

Due to the similar atomic sizes of Zr and Hf, the doping of Hf does not cause serious surface distortion. However, according to Table 1, the shortening of the Hf-Co distance is much greater than that of the Zr-Co distance. This phenomenon demonstrates a very strong interaction between the surface Hf and subsurface Co, which might affect the inward diffusion of oxygen.

### 3.2. O_2_ Molecules Adsorption and Dissociation

**Figure 2:**
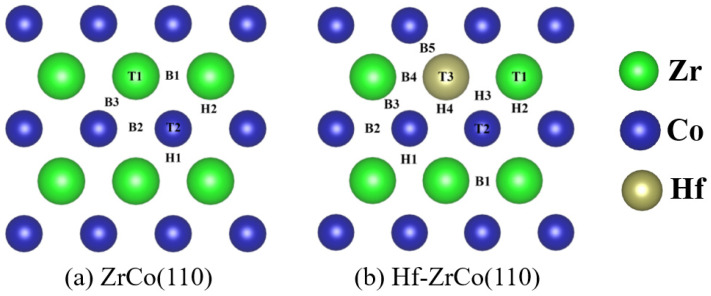
The top views of (**a**) ZrCo(110) surface and (**b**) Hf-ZrCo(110) surface. All possible adsorption sites for oxygen atoms and molecules are marked. (green, blue and brown spheres represent Zr atoms, Co atoms and Hf atoms, respectively).

Figure 2 shows the top views of the ZrCo(110) surface and Hf-ZrCo(110) surface. T1, T2 and T3 represent the top sites of Zr, Co and Hf atoms, respectively. B1, B2, B3, B4, and B5 represent the bridge sites between the atomic neighbors two Zr, two Co, Zr and Co, Hf and Zr, and Hf and Co, respectively. The rest H1, H2, H3, and H4 are the hollow sites formed by two Zr and a Co, two Co and a Zr, a Hf and a Zr, and a Co, and a Hf, and two Co in atoms, respectively. On the ZrCo(110) surface, T3, B4, B5, H3, and H4 sites are missing due to the symmetry.

In our work, we first studied the adsorption of oxygen molecules on the ZrCo(110) surface and the Hf-doped ZrCo(110) surface. For each site, there are three possible adsorption modes for oxygen molecules, namely (a) parallel to the molecular axis on the surface, (b) rotating 90° based on the structure of (a), and (c) perpendicular to the surface. For example, the site at the top of the Zr atom where the oxygen molecule is placed parallel to the X axis is called the T1a site.

Our calculation results in Supplementary Materials (Table S1) show that the adsorption behaviors of oxygen are not significantly affected when using DFT + U methodology. Therefore, DFT + U methodology is not considered in this work.

The adsorption energies and structural parameters of different sites are shown in Table 2. We found that oxygen molecules adsorbed perpendicular to the surface are always not as stable as those adsorbed parallel to the X or Y axis. For example, after structural relaxation, the T1c site adsorption energy is −1.83 eV, while the T1a site adsorption energy is lower (−2.95 eV), which indicates that the adsorption at the T1a site is more stable. Therefore, only adsorption configurations parallel to the X or Y axes are listed in Table 2.

Table 2 shows that the adsorption energies of all sites on the ZrCo(110) surface are negative, which indicates that adsorption at any site is possible. At the T1 and B1a sites, the length of the O–O bond is slightly longer compared to the isolated oxygen molecule bond length (1.2 Å) after adsorption, which indicates that dissociation does not occur. At the T2, B1b, B2, B3, H1, and H2 sites, the O–O distances are 2–3 times longer than those of the isolated oxygen, which indicates that the O–O bonds of oxygen molecules break during the structure relaxation at these sites, completely dissociating into oxygen atoms and subsequently being adsorbed on the ZrCo(110) surface. The adsorption of oxygen molecules at the B3 site is the most stable, with an adsorption energy of −10.14 eV. It can be known that the adsorption sites and directions have significant impacts on the adsorption of oxygen molecules. It is known that both dissociative and non-dissociative adsorptions occur on surfaces, and dissociative adsorption is more favorable from an energy perspective. The more favorable dissociative adsorption was also found on some other active surfaces, like the γ-U(001) surface [30].

Then, the effect of Hf doping on the adsorption behavior of oxygen molecules on the ZrCo(110) surface was studied. Table 2 shows that on the Hf-ZrCo (110) surface, all the adsorption energies are also negative, indicating that any site can be a possible adsorption site. The adsorption energy of oxygen molecules at the T1a site is −2.92 eV, increasing by 0.03 eV compared to that on the ZrCo(110) surface, which means Hf doping slightly reduces the adsorption stability of oxygen molecules at adjacent Zr atoms. And the adsorption energy at the T3a site is more negative (−3.07 eV), indicating that, compared to Zr atoms, the adsorption of oxygen at the top of Hf atoms is more stable. At the T1, T3, B1a, B2a, and B4a sites, the O–O bond length is slightly elongated (1.4–1.5 Å) compared to the isolated oxygen molecule bond length (1.2 Å) after adsorption, which indicates that dissociation does not occur. Oxygen molecules will spontaneously be dissociated at the remaining sites. It can be concluded from Table 2 that there is no significant change in adsorption configuration or adsorption energy after Hf doping; therefore, the Hf doping will not significantly affect the adsorption behaviors of oxygen molecules on the Hf-ZrCo (110) surface.

As shown in Table 2, during structure relaxation, spontaneous dissociation will occur when oxygen molecules are adsorbed at certain sites. In order to study the dissociation characteristics of oxygen molecules, the dissociation barrier of oxygen molecules at the T1a (T3a) site was calculated.

**Figure 3:**
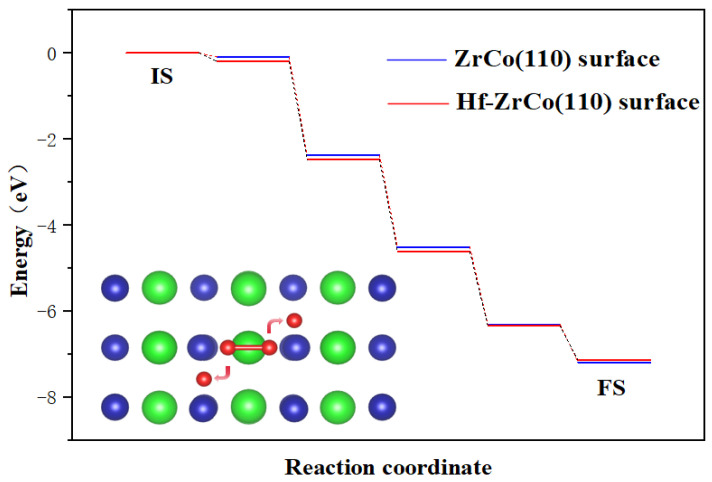
Minimum energy pathway for O_2_ dissociation on the ZrCo(110) surface and Hf-ZrCo(110) surface (IS and FS represent the initial and final states, while green, blue and red spheres represent Zr atoms, Co atoms and O atoms, respectively).

As shown in Figure 3, the dissociation process on the ZrCo(110) surface is exothermic, with a heat release of 7.18 eV, forming a stable structure. There is almost no dissociation barrier, which indicates that the dissociation of oxygen molecules is a spontaneous process, which is in line with the result of Zhang et al. [31]. On the Hf-ZrCo(110) surface, similarly, oxygen molecules undergo spontaneous dissociation, with two oxygen atoms finally stably adsorbed at the H3 site on the surface. That is to say, the doping of Hf will not affect the spontaneous dissociation of oxygen molecules on the ZrCo(110) surface.

### 3.3. O Atoms Adsorption

The adsorption sites of oxygen molecules can be adapted into O atoms. Single O atom adsorption on the ZrCo(110) surface and Hf-doped ZrCo(110) surface was discussed, and the adsorption energies and structure parameters were given in Table 3.

As shown in Table 3, on the ZrCo(110) surface, O atoms cannot be adsorbed stably at the B3 and H2 sites. After structure relaxation, O atoms placed at the B3 and H2 sites will relocate to the H1 and B2 sites, respectively. The adsorption of oxygen atoms at the H1 site is the most stable, with an adsorption energy of −5.01 eV, followed by the B1, B2, T1, and T2 sites, which is consistent with the reported result [31]. In addition, O atom adsorption at all sites belongs to strong chemisorption, which means O atoms can be easily adsorbed on the ZrCo(110) surface.

Similar to the pure surface, the O atoms at B3 and B5 sites on the Hf-ZrCo(110) surface will slide to the H3 site, and the O atoms at H2 and H4 sites will slide to the B2 site. The adsorption of oxygen atoms at the H3 site is the most stable, with an adsorption energy of −5.07 eV. However, there is no obvious difference in adsorption energies and distances of O from the surface between the pure and Hf-doped ZrCo(110) surfaces. The results show that the Hf doping cannot change the adsorption stability of the O atoms on the surface.

**Figure 4:**
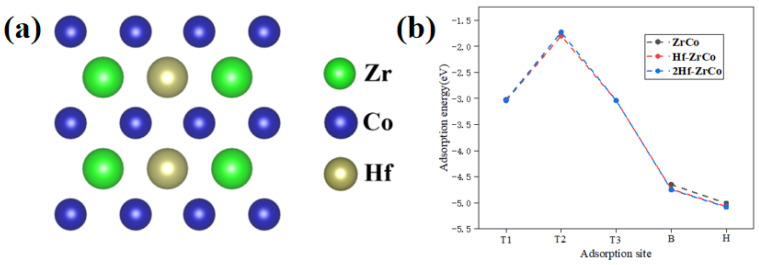
(**a**) The top view of the 2Hf-ZrCo(110) surface, (**b**) O atom adsorption energies on the ZrCo(110), Hf-doped ZrCo(110) and 2Hf-ZrCo(110) surfaces.

To study the concentration of Hf substitution on the O atom adsorption behaviors, as shown in Figure 4a, we built the 2Hf-ZrCo(110) surface by substituting two Zr atoms with two Hf atoms. Figure 4b shows that, compared with the Hf-ZrCo(110) surface, the O adsorption energy at different adsorption sites on the 2Hf-ZrCo(110) surface is only reduced by a range of 0.01–0.07 eV. Therefore, the increasing the Hf doping amount cannot strengthen the adsorption stability of the surface O atoms.

Understanding the permeation of O atoms is also important for acknowledging the mechanism of surface oxidation for ZrCo. O atoms may enter tetrahedral and octahedral sites in the subsurface when diffusing on the surface. As far as the configurations of all tetrahedral sites occupied by oxygen atoms are concerned, after structure relaxation, O atoms slide to the adjacent octahedral sites. O atoms can occupy the octahedral site composed of four Zr atoms and two Co atoms stably, with an adsorption energy of −3.45 eV. However, at the octahedral site composed of four Co atoms and two Zr atoms, O atoms cannot exist stably. After structure relaxation, O atoms will relocate to the surface or second-layer surface. Although the O atom can stably occupy the octahedral sites, the adsorption energies at the octahedral sites are smaller than those at the surface adsorption sites. Therefore, the O atoms are more inclined to be adsorbed on the ZrCo surface than the subsurface.

On the Hf-ZrCo(110) surface, O cannot occupy the tetrahedral sites and will move to the adjacent octahedral sites. The O atoms can stably occupy the octahedral site composed of four Zr atoms and two Co atoms, with an adsorption energy of −3.43 eV, and they can also stably occupy the octahedral site composed of three Zr atoms, one Hf atom, and two Co atoms, with an adsorption energy of −3.44 eV.

Based on the analysis of the adsorption energy of the O atoms, some conclusions can be drawn that are applicable to both the ZrCo(110) surface and the Hf-doped ZrCo(110) surface as well: 1. The hollow sites are the most stable adsorption sites for O atoms on the surface. 2. Compared to the tetrahedral and octahedral sites on the subsurface, the O atoms are most likely to be adsorbed on the surface. 3. Tetrahedral sites are not stable adsorption sites for O atoms, and the internal diffusion of the O atoms is carried out through the octahedral sites.

### 3.4. Diffusion of Oxygen

**Figure 5:**
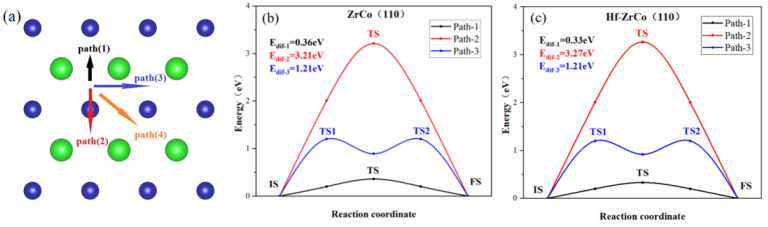
(**a**) The paths and minimum energy pathway for O diffusion on (**b**) ZrCo(110) surface and (**c**) Hf-doped ZrCo(110) surface.

The previous section shows that the H1(H3) site is the most stable O atom adsorption site. Oxygen atoms may diffuse to their neighboring stable H1(H3) sites in different paths. We calculated all four possible diffusion paths, as shown in Figure 5a, to study the O diffusion behavior. The calculation results indicate that path(3) and path(4) have the same diffusion paths; therefore, only the minimum energy paths of path(1): H1-B1-H1(H3-B4-H3), path(2): H1-T2-H1(H3-T2-H3), and path(3): H1-B3-B2-B3-H1(H3-B5-B2-B5-H3) are shown in Figure 5b,c.

As shown in Figure 5, on the ZrCo(110) surface, the O atom diffusing across the top of the Co atom has the largest diffusion energy (E_dif_), which is much higher than other paths, reaching up to 3.21 eV, while path(1), across the bridge of two Zr atoms, requires the lowest energy of 0.36 eV.

On the Hf-ZrCo(110) surface, path(2) across the top of the Co atom still requires the highest diffusion energy, with the E_dif_ of 3.27 eV. Similarly, path(1) across the bridge of Zr and Hf, has the lowest E_dif_ of 0.33 eV, slightly lower than the diffusion energy on the ZrCo(110) surface. Therefore, the doping of Hf has few effects on the diffusion of O atoms on the surface.

It is known that O atoms may enter tetrahedral and octahedral sites in the subsurface when diffusing on the surface. In this section, we choose the O atom adsorption at the H1/H3 site as the initial state (IS) because they are the most stable configurations. Similarly, O adsorption at the most stable octahedral hole in the subsurface is selected as the final state.

**Figure 6:**
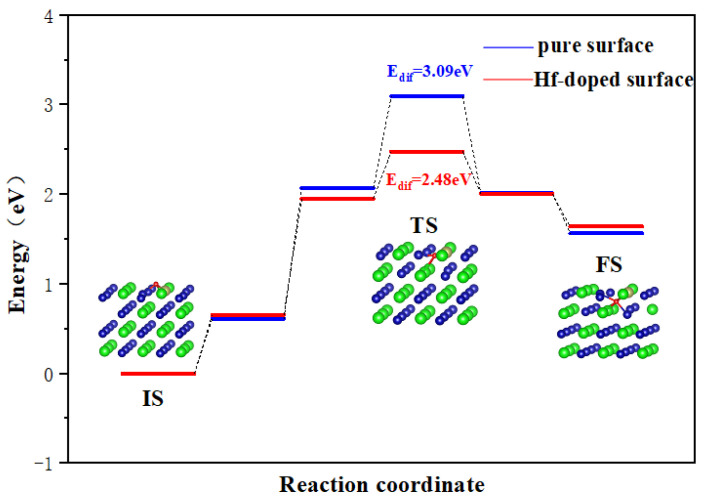
Minimum energy pathway for O inward diffusion on the ZrCo(110) surface and Hf-doped ZrCo(110) surface.

As shown in Figure 6, for a pure ZrCo(110) surface, the E_dif_ for O to diffuse inward to the subsurface is 3.09 eV. As discussed above, the lowest E_dif_ for O atom diffusion on the surface is only 0.36 eV, which is far lower than the inward diffusion barrier. It can be safely concluded that the oxygen atoms will preferentially occupy the surface and then diffuse to the subsurface.

On the Hf-doped ZrCo(110) surface, the E_dif_ for O to diffuse inward to the subsurface is 2.48 eV. This result shows that the doping of Hf reduces the E_dif_ for O to diffuse inward to the subsurface by 19.7%. That is to say, the doping of Hf enhances the diffusion ability of O atoms, which is obviously unfavorable to their resistance to surface oxidation. The most stable (110) surface cannot resist surface oxidation, let alone other planes. The reason why Hf does not enhance the resistance to O_2_ poisoning of ZrCo alloys like ZrV_2_ alloys may be due to their different crystal structures and chemical properties.

### 3.5. Inward Diffusion of Hydrogen

The permeation of the H atom from the surface into the bulk is one of the key steps in hydrogen storage. To study the effect of O adsorption and Hf doping on the H inward diffusion behaviors, we calculated the inward diffusion processes of a H atom to the subsurface on the pure ZrCo(110) surface, the O adsorbed ZrCo(110) surface, and the O adsorbed Hf-doped ZrCo(110) surfaces, respectively.

**Figure 7:**
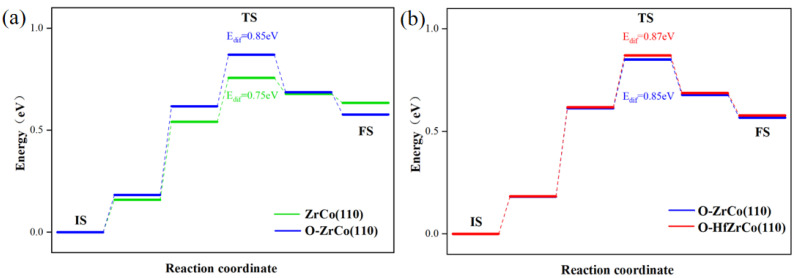
Minimum energy pathway for H inward diffusion on (**a**) ZrCo(110) and O-adsorbed ZrCo(110) surfaces; (**b**) O-adsorbed Hf-doped ZrCo(110) surfaces.

As shown in Figure 7a, the diffusion barrier from the pure ZrCo(110) surface to the subsurface is 0.75 eV, which is in line with the reported results (0.79 eV) [22]. As for the O-adsorbed ZrCo(110) surface, the hydrogen atom has to overcome an additional 0.1 eV energy barrier to diffuse inward to the subsurface. The existence of O on the ZrCo(110) surface inhibits H inward diffusion.

Figure 7b shows that after Hf doping, the H inward diffusion energy on the O-adsorbed ZrCo(110) reaches 0.87 eV, increasing by 2.3%, implying that Hf doping slightly suppresses the inward diffusion of hydrogen. Thus, Hf doping might do harm to the hydrogen storage properties of ZrCo alloys in an oxygen-containing environment to a degree.

### 3.6. Partial Density of State

In this work, to study the interactions between oxygen and surface atoms from the point of view of electronic structure, the partial densities of states (PDOS) of the O_2_ adsorption at T1a/T3a sites and the O adsorption states at H1/H3 sites are calculated.

**Figure 8:**
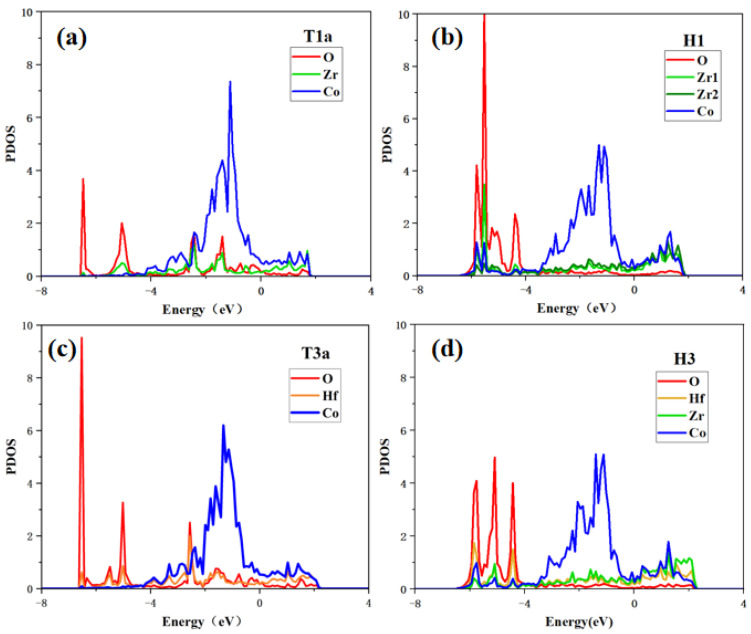
Partial density of states of (**a**) O_2_ adsorption at T1a site, (**b**) O adsorption at H1 site on the ZrCo(110) surface, (**c**) O_2_ adsorption at T3a site, and (**d**) O adsorption at H3 site on the Hf-ZrCo(110) surface.

As shown in Figure 8, when O_2_ molecules are adsorbed at the T1a site, O and Zr have high PDOS overlaps in the range of −6.4 eV to 2.0 eV, indicating that there is a strong bonding tendency between O and Zr. While the electrons of the adjacent Co and O have few overlaps, indicating that O tends to bond with Zr rather than Co. For O atom adsorption at the H1 site, the peaks of O and Zr or Co are well overlapped in the range of −6.4 eV to −4.0 eV, which indicates the significant hybridization between the O atom and the adjacent atoms, fitting the lowest adsorption energy shown in Table 3. In addition, it can be found that the O atom unequally interacts with the Zr and Co atoms because the PDOS overlap between O and Zr is larger than that between O and Co atoms. These results show that the bonding of O with Zr at whatever site is stronger than that of O with Co.

For O_2_ molecules adsorption at the T3a site on the Hf-ZrCo(110) surface, the hybridization of O and Hf is obvious in the range of −6.4 eV to 2.0 eV. As for O atom adsorption at the H3 site, O is orbital hybridized with the adjacent Hf, Zr, and Co in the range of −6.4 eV to −4.0 eV. It is worth noting that the PDOS overlaps between O and Hf atoms on the doped surface are slightly higher than the corresponding overlaps between O and Zr atoms on the pure surface; that is, the bonding strength between O and Hf atoms on the doped surface is stronger than that between O and Zr atoms on the pure surface. Therefore, the O atom is more stable at the H3 site on the doped surface than at the H1 site on the pure surface, which is in line with the previous results that the adsorption energy of O atoms at the H3 site is more negative.

### 3.7. Differential Charge Density

The differential charge density displays the charge transfer between oxygen and its surrounding atoms, helping us analyze the interaction between these atoms. The differential charge density can be calculated by removing the surface charge density (ρ_surf_) and the charge density of O_2_ molecules or O atoms(ρ_O_) from the total charge density of the adsorption system(ρ_total_), which is described by the following formula:

ρ_dif_ = ρ_total_ − ρ_surf_ − ρ_O_ (3)

**Figure 9:**
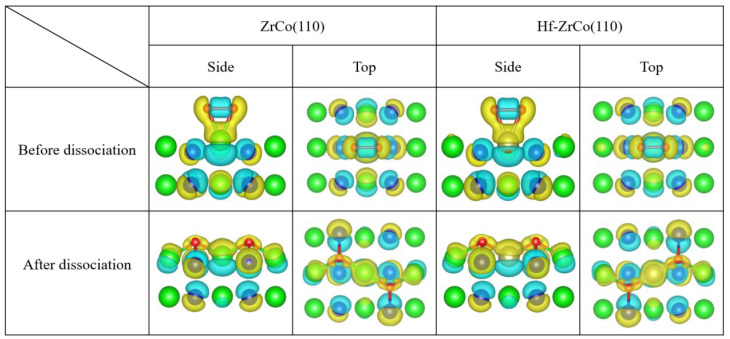
The differential charge density of O adsorption at T1/H1 site on the ZrCo(110) surface and O adsorption at the T3/H3 site on the Hf-ZrCo(110) surface (the yellow region represents the charge accumulation, while the blue region represents the charge depletion).

Figure 9 shows the top view and side view of the differential charge density of oxygen molecules on the ZrCo(110) and the Hf-doped ZrCo(110) surfaces before and after dissociation. It can be seen that when O_2_ molecules are adsorbed at the T1 site on the pure ZrCo(110) surface in molecular form, the two O atoms still exhibit covalent interaction. In addition, there is a larger charge exchange region between O and the adjacent Zr, and O tends to obtain electrons while Zr loses electrons, indicating the strong interaction between O and Zr. When the dissociated O atoms are adsorbed at the H1 site, the charge accumulation region is distributed around the O atoms, and the charge depletion area is distributed among the neighboring two Zr atoms and one Co atom. Therefore, there is a strong interaction between the oxygen atom and its neighboring atoms. The charge accumulation region of O is more inclined towards the Zr atom, indicating that Zr provides more charge to the O atom than Co. Meanwhile, the more charge exchange implies that O adsorption at H1 site is the most stable, which is compatible with the O adsorption behavior analysis results.

For the Hf-ZrCo(110) surface, when O_2_ molecules are adsorbed at the T3 site in molecular form, the two O atoms still exhibit covalent interaction, and the evident charge transfer from Hf to O shows the strong interaction between O and the doped Hf. When the dissociated O atoms are adsorbed at the H3 site, the O charge accumulation area is closer to the adjacent Hf and Zr, and the bonding tendency between Zr/Hf and O is higher than that of Co. However, the region of charge exchange does not significantly expand or decrease, which is consistent with the conclusion that Hf doping will not have a significant impact on O adsorption behavior.

### 3.8. Bader Charge Analysis

Bader charge can be used to quantitatively study the charge transfer, which can help us further understand the interaction between oxygen and its surrounding atoms before and after dissociation.

**Figure 10:**
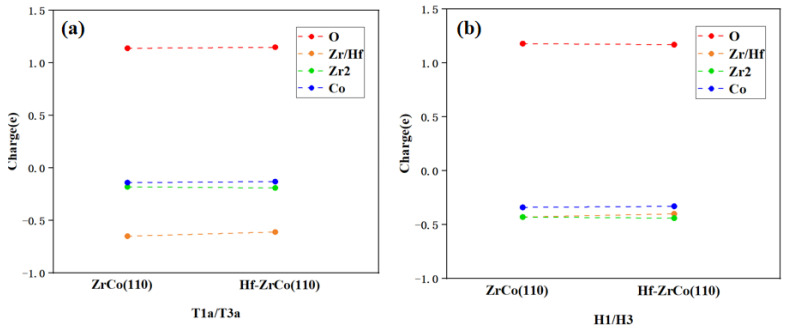
Charge exchange (**a**) before and (**b**) after O_2_ dissociation on ZrCo(110) and Hf-ZrCo(110) surfaces.

As shown in Figure 10, on the ZrCo(110) surface, when an oxygen molecule is adsorbed at the T1a site, the oxygen molecule can obtain 1.14e from the surface, with 57% of the charge coming from the closely surrounded Zr atoms. This result shows that the interaction between O and Zr is much stronger than that between O and other adjacent atoms. After Hf replaces Zr, Hf provides 53% of the charge to the O atom, indicating that O mainly interacts with the doped Hf instead of the surrounded Zr and Co atoms.

On the ZrCo(110) surface, when an oxygen atom is adsorbed at the H1 site, a single oxygen atom can obtain 1.18 e from the surface. The higher charge obtained shows that the oxygen atom adsorbed at the H1 site is more stable than the oxygen molecule adsorbed at the T1a site. In addition, 36% of the charge obtained by O is provided by one adjacent Zr atom, and only 28% of the charge is provided by the adjacent Co atom, which indicates the bonding of O and Zr is stronger than that of O and Co. After Hf doping, Hf provides 34% of the charge to O, which is similar to Zr. Generally speaking, the Bader charge analysis shows that Hf doping has no significant effect on the charge transfer of the surface atoms, which is in line with the previous analyses.

## 4. Conclusions

In this study, we observed that on the pure ZrCo(110) surface, the adsorption and dissociation of oxygen molecules occur readily, with dissociative adsorption being energetically favored. The introduction of Hf as a dopant has minimal effect on oxygen adsorption and dissociation behavior. Oxygen atoms are found to be stably absorbed at the H1 site on the ZrCo(110) surface. As to the adsorption in the subsurface, compared with the tetrahedral sites, the octahedral sites are more favorable to adsorb the oxygen molecules. However, due to their higher adsorption energy, the O atoms are more inclined to be adsorbed on the ZrCo surface than on the subsurface. The diffusion behavior of oxygen on the pure ZrCo(110) surface reveals that oxygen tends to fill the surface before diffusing inward. However, the diffusion energy barrier is reduced after Hf doping, which diminishes the resistance to surface oxidation. Furthermore, the analysis of electronic structure and charge transfer shows no significant weakening of the bonding interactions between O and its surrounding atoms after the Hf doping. Therefore, our study suggests that the doping of Hf is not a recommended approach to improving the resistance to O_2_ poisoning, although it was reported that Hf doping is beneficial for improving the anti-disproportionation of ZrCo.

## Figures and Tables

**Table 1:** The z coordinates of atoms on the ZrCo(110) surface and Hf–ZrCo(110) surface.

Layer	ZrCo(110)			Hf-ZrCo(110)		
	Before Relaxation	After Relaxation	Δd	Before Relaxation	After Relaxation	Δd
1st (Zr)	6.775	6.769	−0.08	6.775	6.760	−0.22
1st (Co)	6.775	6.478	−4.38	6.775	6.474	−4.44
1st (Hf)	-	-	-	6.775	6.709	−0.97
2nd (Zr)	4.517	4.509	−0.18	4.517	4.504	−0.29
2nd (Co)	4.517	4.590	1.61	4.517	4.575	1.28
d_12_(Zr-Co)	2.258	2.179	−3.50	2.258	2.185	−3.23
d_12_(Co-Zr)	2.258	1.969	−12.80	2.258	1.970	−12.75
d_12_(Hf-Co)	-	-	-	2.258	2.134	−5.49

**Table 2:** O_2_ adsorption energy(E_ads_) and O-O distance(d_O-O_) for the ZrCo(110) surface and Hf-doped ZrCo(110) surface.

Sites	ZrCo		Hf-ZrCo	
	E_ads_	d_O-O_	E_ads_	d_O-O_
T1a	−2.95	1.480	−2.92	1.479
T1b	−2.91	1.488	−2.89	1.486
T2a	−7.93	3.533	−7.83	3.460
T2b	−9.66	3.021	−9.74	3.040
T3a	-	-	−3.07	1.493
T3b	-	-	−3.01	1.501
B1a	−3.59	1.452	−3.36	1.451
B1b	−8.77	2.460	−8.79	2.477
B2a	−4.01	1.455	−4.02	1.456
B2b	−8.77	2.461	−8.79	2.459
B3	−10.14	3.727	−10.16	3.727
B4a	-	-	−3.66	1.460
B4b	-	-	−8.81	2.436
B5	-	-	−10.21	3.727
H1	−9.88	4.517	−9.95	4.540
H2	−9.99	3.277	−10.01	3.276
H3	-	-	−9.95	4.495
H4	-	-	−10.08	3.201

**Table 3:** The adsorption energies (E_ads_) and the distances of O from the surface for the ZrCo(110) surface and Hf-doped ZrCo(110) surface.

Sites	ZrCo			Hf-ZrCo		
	E_ads_	d_o-surf_	Note	E_ads_	d_o-surf_	Note
T1	−3.04	2.18	-	−3.02	2.19	
T2	−1.80	1.79	-	−1.80	1.79	
T3	-	-	-	−3.04	2.09	
B1	−4.65	1.32	-	−4.66	1.32	
B2	−4.12	0.09	-	−4.15	0.10	
B3	−5.01	1.00	to H1	−5.07	0.99	to H3
B4	-	-	-	−4.74	1.29	
B5	-	-	-	−5.07	1.00	to H3
H1	−5.01	1.00	-	−5.02	0.99	
H2	−4.12	0.09	to B2	−4.15	0.10	to B2
H3	-	-	-	−5.07	0.99	
H4	-	-	-	−4.10	0.10	to B2

## Data Availability

The original contributions presented in the study are included in the article/Supplementary Material, further inquiries can be directed to the corresponding author.

## Supplementary Materials

The following supporting information can be downloaded at: https://www.mdpi.com/article/10.3390/ma17102424/s1, Table S1. O_2_ adsorption energy (Eads) and O-O distance (d/_O-O_) on the ZrCo(110) and Hf-ZrCo(110) surfaces.
