# Supplementary material for: Influence of Hf Doping on the Oxygen Behaviors on ZrCo(110) Surface Using First-Principles Calculation
Source: Materials (Basel). 2024 May 17;17(10):2424. doi: 10.3390/ma17102424 (PMC11123304; doi:10.3390/ma17102424)
Supplement: Supplementary file 1 [file materials-17-02424-s001.zip › materials-2931523-supplementary.pdf]

materials

MDPI

Supplementary Materials

Influence of Hf Doping on the Oxygen Behaviors on ZrCo(110) Surface Using First-Principles Calculation

Ruijun Qian [1], Habibullah [2], Meitong Ye [1], Wanglai Cen [2] and Chaoling Wu [1,2,*]

1 College of Materials Science and Engineering, Sichuan University, Chengdu 610064, China; qrj1430757297@163.com (R.Q.); 18328300781@163.com (M.Y.)
2 Institute of New-Energy and Low-Carbon Technology, Sichuan University, Chengdu 610064, China; habibullah@stu.scu.edu.cn (H.); cenwanglai@scu.edu.cn (W.C.)
* Correspondence: wuchaoling@scu.edu.cn; Tel.: +86-18980672160

Table S1. O_2 adsorption energy (Eads) and O-O distance ($d/_{O-O}$) on the ZrCo(110) and Hf-ZrCo(110) surfaces.

pproach	ZrCo-B3		Hf-ZrCo-B5	
	E_{ads}(eV)	d_{O-O}(Å)	E_{ads}(eV)	d_{O-O}(Å)
DFT	-10.14	3.73	-10.21	3.64
DFT+U	-10.64	3.65	-10.78	3.56
